# Stereoscopic Vision in the Absence of the Lateral Occipital Cortex

**DOI:** 10.1371/journal.pone.0012608

**Published:** 2010-09-07

**Authors:** Jenny C. A. Read, Graeme P. Phillipson, Ignacio Serrano-Pedraza, A. David Milner, Andrew J. Parker

**Affiliations:** 1 Institute of Neuroscience, Newcastle University, Newcastle upon Tyne, United Kingdom; 2 Institute of Adaptive and Neural Computation, School of Informatics, Edinburgh University, Edinburgh, United Kingdom; 3 Department of Psychology, Durham University, Durham, United Kingdom; 4 Department of Physiology, Anatomy and Genetics, Oxford University, Oxford, United Kingdom; National Institute of Mental Health, United States of America

## Abstract

Both dorsal and ventral cortical visual streams contain neurons sensitive to binocular disparities, but the two streams may underlie different aspects of stereoscopic vision. Here we investigate stereopsis in the neurological patient D.F., whose ventral stream, specifically lateral occipital cortex, has been damaged bilaterally, causing profound visual form agnosia. Despite her severe damage to cortical visual areas, we report that DF's stereo vision is strikingly unimpaired. She is better than many control observers at using binocular disparity to judge whether an isolated object appears near or far, and to resolve ambiguous structure-from-motion. DF is, however, poor at using relative disparity between features at different locations across the visual field. This may stem from a difficulty in identifying the surface boundaries where relative disparity is available. We suggest that the ventral processing stream may play a critical role in enabling healthy observers to extract fine depth information from relative disparities within one surface or between surfaces located in different parts of the visual field.

## Introduction

**Figure 1:**
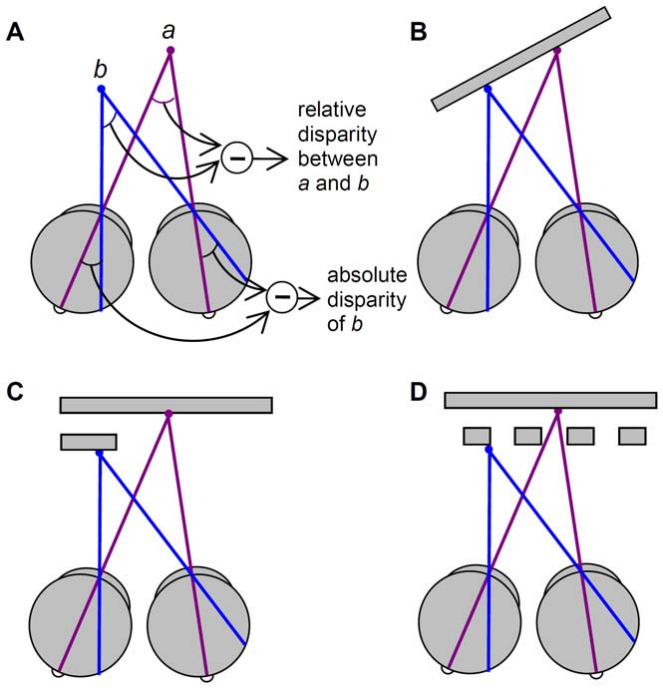
Different stimulus configurations that give rise to relative disparity. **A**: Relative disparity between two points. Absolute disparity of an object = difference in its angular distance from the fovea in the two eyes. Here the eyes are looking at point *a*, so *a* has zero absolute disparity but *b* has a non-zero value. The relative disparity of the two points is equal to the difference in their absolute disparities (equivalent to difference in angles subtended at each point, as shown). **B**: Relative disparity between different points on a surface. **C**: Relative disparity between surfaces, one of which occludes the other. **D**: Relative disparity between transparently-visible surfaces, as can occur where a non-opaque surface or structure, such as a picket-fence or tree, partially occludes a more distance surface.

Humans use the different viewpoints provided by their two eyes to produce a vivid percept of the world in depth. A distinctive feature of stereoscopic perception is that we are far better at judging the relative disparity between two features than judging the absolute depth of an isolated feature [1]. For example, in Figure 1A, the eyes are directed at point *a*, so the retinal images of point *b* lie further from the fovea in the right eye than in the left. This difference between the retinal images of point *b* is called an absolute disparity. If the absolute disparity is large enough, we will perceive *b* as closer to us than the fixation point, even if *b* is presented in isolation. However, our sensitivity to the depth of *b* is greatly enhanced if points *a* and *b* are simultaneously visible, so that we can directly compare the *relative disparity* between the two points. In a complex scene, one can distinguish many different sorts of relative disparity [2]: e.g. relative disparity within a surface (Figure 1B), between adjacent surfaces (Figure 1C), and between surfaces viewed transparently (Figure 1D).

The cortical pathways underlying our sensitivity to these various forms of relative disparity are unclear. Area V2 contains neurons tuned to adjacent-surface relative disparity [3]–[5], and the proportion of such neurons increases along the ventral stream [6]–[8]. However, dorsal areas V3A and the caudal intraparietal sulcus CIPS are also strongly activated by adjacent-surface relative disparity [9]. Human fMRI reveals no difference in the response of V1 and V2 to the relative disparity between transparent surfaces; the ventral stream responds to both absolute and transparent-surface relative disparity while dorsal areas respond mainly to absolute disparity [10].

One particular stimulus that has revealed intriguing differences between the ventral and dorsal streams is the anti-correlated random-dot stereogram, in which the colours of black and white dots are switched over in one eye with respect to the other. Such a stimulus does not correspond to any real physical surface, so a system designed to detect the depths of real surfaces should not respond to it [11], and in accordance with this expectation, neither humans nor monkeys can discriminate depth in such stimuli [12]–[15]. A naïve disparity detector based on cross-correlating the two eyes' images will respond, but with a sign inversion [16]. This signature sign-inversion is found in V1 [14], in many neurons in MT [17], in disparity filters deduced from human psychophysics [18], and in rapid vergence corrections with anti-correlated stimuli [19].

Beyond V1, it has been suggested that ventral and dorsal streams may differ in their responses to anti-correlated stimuli [2]. Dorsal areas MST [20] and MT [17] contain many neurons which are tuned to disparity in anti-correlated stimuli, whereas in ventral areas V4 [21] and IT [22], most neurons are not tuned to anti-correlated disparity. In fMRI, Bridge & Parker [23] found that the largest differences in activation between correlated vs anti-correlated stimuli alternating in depth are found in areas MT and LO. Preston et al. [24] performed a multi-voxel pattern analysis to estimate how much information the BOLD signal in different areas contains about the sign of disparity in correlated and anti-correlated stimuli. They found that, in ventral areas V3v and V4, disparity sign could be read out with about the same accuracy for both correlated and anti-correlated stimuli, whereas in LO and also in all dorsal areas examined, much greater accuracy was obtained for correlated stimuli. Thus, all these different studies agree that the strongest and most reliable differences between correlated and anti-correlated stimuli are found in the ventral stream.

These neurophysiological and imaging studies can only reveal correlations between brain activity and behaviour. Acquired brain lesions provide one of the few available ways of testing for causal relationships between brain circuitry and behaviour in humans. Here we present the first neuropsychological study to investigate the cortical processing of relative disparity. In 1988, Patient DF suffered carbon monoxide poisoning, which generated bilateral damage to the lateral occipital cortex, an important component of the ventral stream [25], [26]. As a result, DF has profound visual form agnosia. Although she has partial visual experience of the world, she is unable to recognize objects by shape, or even to report correctly whether a line or grating is vertical or horizontal [25]–[27].

The initial description of DF (Milner et al. 1991) reported that she retains stereo vision (though unable to identify the shape that is present in depth, and with lower acuity than controls). Beyond that, however, there has been little systematic study of her depth perception. Here, we present a detailed examination of DF's stereoscopic vision, including all three forms of relative disparity identified in Figure 1.

## Methods

### Experimental stimuli

**Figure 2:**
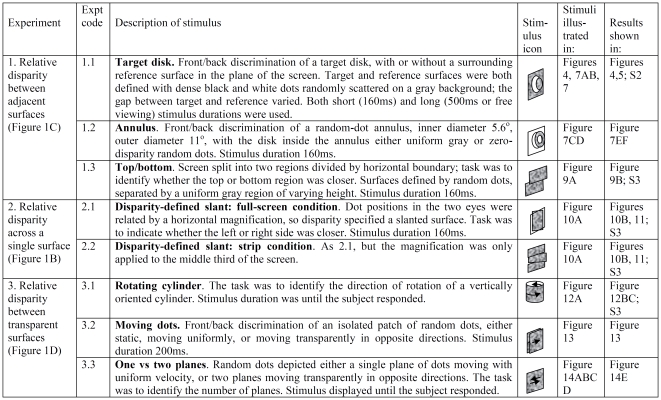
Reference list of experiments. The experiments are grouped together according to the type of relative disparity investigated, and each stimulus is given a numerical identifier. For experiments which were performed at different stimulus durations, the suffix L indicates long duration (either 500ms or until the subject responded) and S indicates short duration (stimulus on-screen for 160ms or 200ms, too briefly for saccades or vergence movements). Figure 3 shows when DF was tested on each stimulus.

**Figure 3:**
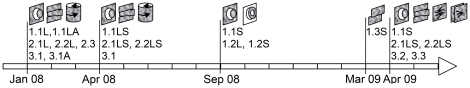
Timeline of experiments performed by DF. See Figure 2 for experiment codes. For experiments done at both short and long durations, the duration is indicated by the suffix S or L. The suffix A indicates the anti-correlated version of the stimulus.

This paper describes a sequence of experiments, designed to probe different aspects of DF's stereo vision, carried out on five occasions over a period of some 15 months. As we learnt more about DF's abilities, we adapted the stimuli accordingly and introduced new stimuli to exclude various interpretations. The experiments will be described in detail as each is introduced but they are summarized in Figure 2, with each stimulus given a numerical identifier. The duration of each stimulus is also specified in Figure 2. Experiments performed at long (500ms or free viewing) and short (160ms or 200ms) durations are indicated by the suffix *L* or *S* in Figure 2. Some experiments were performed with anti-correlated stimuli. These are stimuli in which the contrast polarity of one eye's image is inverted, so that black pixels are replaced with white and vice-versa. These are indicated by the suffix *A* added to the stimulus identifier. Figure 3 provides a timeline showing when DF was tested on each stimulus.

For each stimulus, we initially explained the task to the participant by presenting a few examples in which we described the correct response. During data collection, no feedback on individual trials was provided to participants.

### Subjects

Patient DF was aged 53–55 at the times of testing. DF's brain damage has been assessed by structural and functional MRI measurements [25], [26]. The principal lesions in visual cortex lie bilaterally in the lateral occipital region, specifically area LO, though there is diffuse damage elsewhere, as is typical following carbon monoxide poisoning. The lateral occipital area is clearly compromised on structural scans and is not activated functionally during scans that show a greater activation to coherent, versus scrambled, visual objects in control subjects [26].

We compared DF's performance with that of 7 controls: 2 authors (JR, female, aged 35 years at the time of testing; GP, male, 29) and 5 volunteers without previous psychophysical experience, of similar age to DF: three females, F1 (53), F2, (51), F3 (64), and two males, M1 (60), M2 (63). DF wore prescription spectacles suitable for the viewing distance of 90cm, and controls wore their normal visual correction. Psychophysical experiments were carried out at Durham, Oxford and Newcastle Universities with the understanding and written consent of each subject, and the study complies with the Code of Ethics of the World Medical Association (Declaration of Helsinki). Psychophysics research was approved by internal ethics committees at each institution where experiments were conducted.

### Equipment

All experiments were programmed using *Matlab* (The Mathworks, Natick, MA) with the Psychophysics Toolbox [28], [29] running on a PC. Stereo images were presented in red/blue anaglyph. Except for Experiment 1.3, stimuli were presented on an *Iiyama* flat-screen LCD monitor, 1280×1024 pixels and 37.5×30cm, viewed at 90cm in normal ambient lighting. 1 pixel subtended 0.02° and the whole display subtended 23.5° horizontally and 18.9° vertically. In Experiment 1.3 only, stimuli were presented on a CRT monitor, 1280×1024 pixels and 30×24cm, viewed at 53cm in a dark room. All our experiments used random-dot patterns. These enable the binocular disparity depth cue to be isolated from other cues which signal depth in natural viewing, such as convergence, occlusion, size, texture etc [30]. The random-dot patterns consisted of equal numbers of black and white dots on a gray background with the same mean luminance. The dots were either 7 or 9 arcmin across. Their density was such that if none of the dots overlapped, they would have occupied 30% of the stimulus area. Anti-aliasing was used to produce sub-pixel displacements. In between stimuli, observers converged their eyes on a fixation cross in the plane of the computer screen. Nonius lines were not used, since this would add to the complexity of the tasks DF was being asked to carry out, and since it seemed unlikely that a patient with visual form agnosia would be able to monitor vergence using Nonius lines.

### Data analysis

Psychometric functions were fitted with a cumulative Gaussian scaled to run from 0.5% to 99.5%, i.e. allowing for a 1% lapse rate:
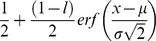
(1)where *l* = 0.01 is the lapse rate, μ is the point of subjective equivalence, σ is the threshold, and *x* is the metameter, i.e. either disparity or magnification. The fitted threshold was not significantly affected by the precise value of the lapse rate, but removing the lapse rate altogether produced artefactually large thresholds for a few psychometric functions (see Supporting Information Files S1 and [31]). Parameters were adjusted, using MATLAB's FMINSEARCH function, to maximize the likelihood.

In both the main paper and the Supporting Information, error bars show the 95% confidence intervals. For psychometric data, these were calculated using the score confidence interval for simple binomial statistics [32]. Other error bars were obtained by bootstrap resampling [33]. First, a new set of simulated psychophysical data was generated from the original cumulative Gaussian fit [34]. Each set of simulated data was then analysed in exactly the same way as the original data-set, in order to produce a different estimate of the quantity of interest (e.g. threshold, regression gradient). This process was repeated 10,000 times, and the 2.5 and 97.5 percentiles were taken as the end-points of the 95% confidence interval on the original quantity.

To assess whether DF's performance differed significantly from those of controls, we used the techniques developed by Crawford & Garthwaite [35], [36] for the analysis of neuropsychological data with single cases, and implemented in their computer programs SINGLIM.EXE and SINGSLOPE.EXE (available online at http://www.abdn.ac.uk/~psy086/dept/SingleCaseMethodsComputerPrograms.HTM).

## Results

### Experiment 1: Sensitivity to absolute and relative disparity between frontoparallel surfaces

#### Experiment 1.1: Depth discrimination of a target disk

**Figure 4:**
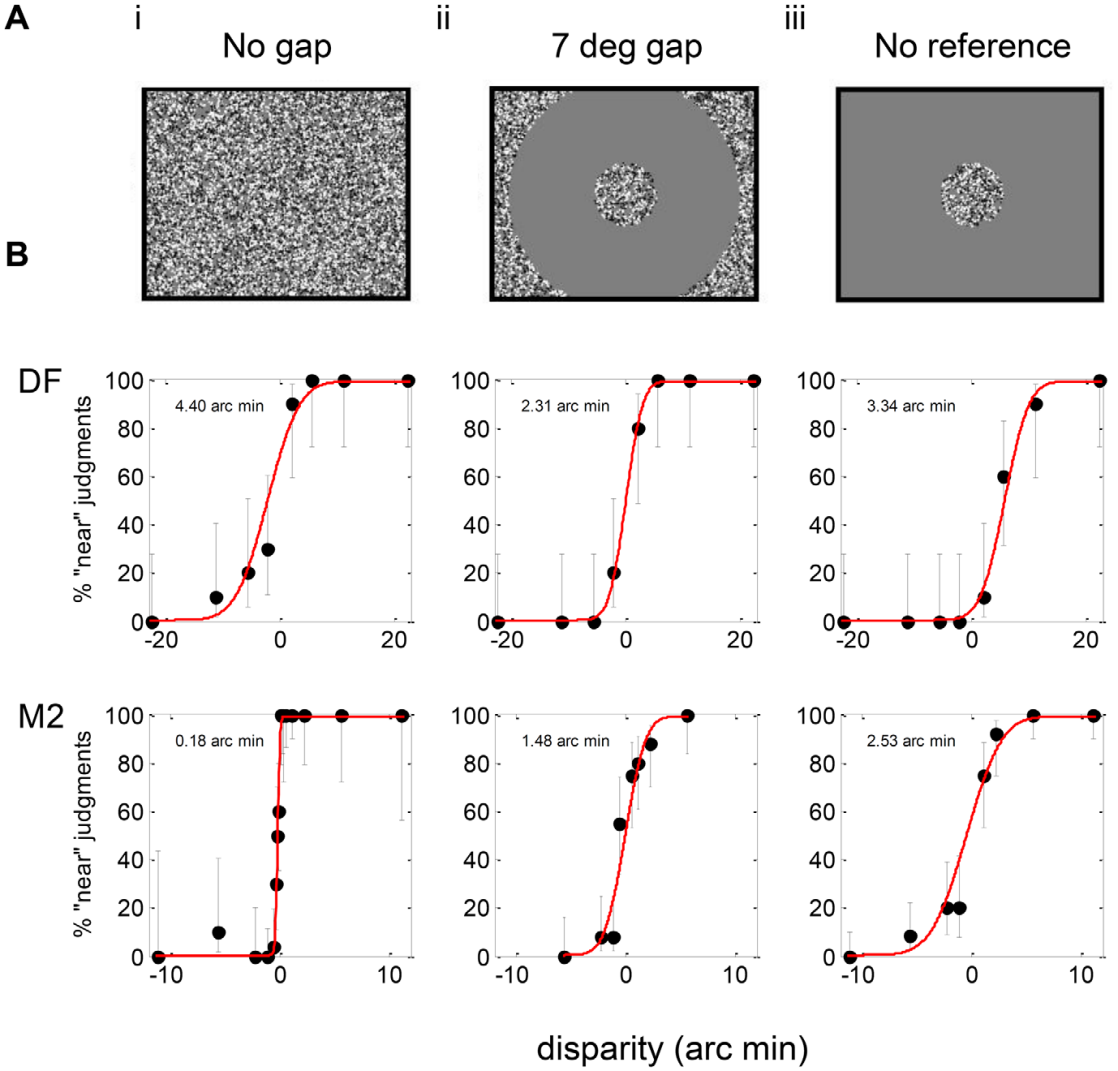
Experiment 1.1*L*: Front/back discrimination on a target disk. **A:** Stimuli: (i) Central disparate disk is contiguous with a zero-disparity random-dot reference surface; (ii) central disk separated from reference surface by annulus of width 7°; (iii) no reference surface. In each case, the central target was 5.6° in diameter. **B:** Psychometric functions for patient DF and age-matched control M2, showing percentage “far” judgements as a function of target disparity, for long stimulus presentations. See Supporting Information File S1 for full data.

In Experiment 1.1, observers viewed a random-dot stereogram that depicted a disparate disk, 5.6° in diameter, with a surrounding reference surface in the plane of the screen (Figure 4Ai). They reported whether the disk appeared in front of or behind the screen. This stimulus presents both absolute and relative disparity to the observer: the absolute disparity of the central disk compared to the binocular fixation adopted by the subjects (typically the fixation cross displayed initially), and the relative disparity between the central disk and the zero-disparity surround. Next, the experiment was repeated with a featureless, annular gap between the disk and the reference surface (Figure 4Aii), and finally, if the subject was still able to do the task, the disk was presented on a featureless gray background with only the edges of the display screen available as a reference for relative depth (Figure 4Aiii). This stimulus presents the same absolute disparity as the first, but the information about relative disparity is greatly reduced. This enables us to quantify the additional sensitivity contributed by adjacent-surface relative disparity.

**Figure 5:**
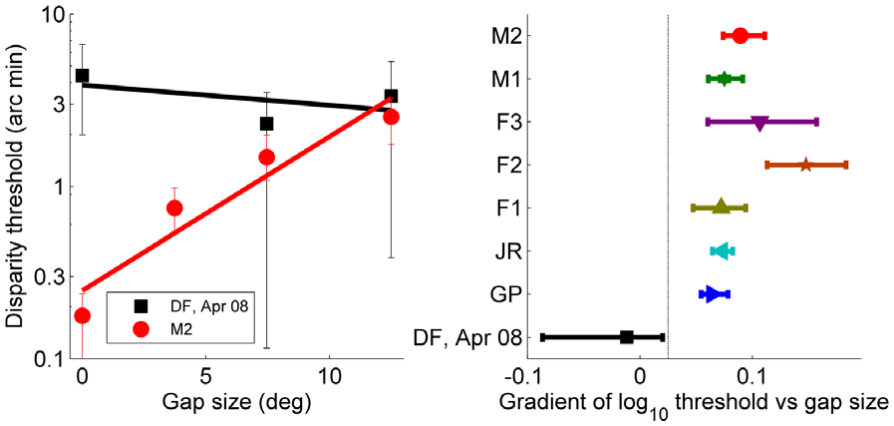
Results of Experiment 1.1*L*, long stimulus duration. **A:** Threshold as function of gap size for DF (black squares, gray diamonds) and M2 (red circles), together with regression lines for log threshold as a linear function of gap size. **B:** Regression gradients (log_10_ arcmin/deg) for DF (black squares) and 7 controls (colored symbols). For the regressions, the “no reference” condition was taken to be equal to a gap of 12.5°. This is the smallest annulus width that completely removes the reference surface from view, even in the diagonal corner of the screen. In both panels, the error-bars are 95% confidence intervals generated by bootstrap resampling. The vertical dashed line in panel B demonstrates that the 95% confidence interval for DF's gradient does not overlap the 95% confidence interval of any of the 7 controls. See Supporting Information File S1 for full data.

Figure 4B shows psychometric functions for patient DF and for a typical control subject M2, for a stimulus duration of 500ms. DF's thresholds are higher than M2's and, critically, they are essentially similar for all 3 gaps, whereas M2's thresholds increase by an order of magnitude as gap size increases. This is shown in Figure 5A, where thresholds are plotted on log axes as a function of gap size (the “no reference” condition is plotted as a gap of 12.5°, which is the smallest gap that completely removes the reference surface from view, even in the diagonal corner of the screen).

To quantify this across subjects, we calculated the gradient of a regression line fitted to log_10_(threshold) as a function of gap size. Figure 5B shows this for DF and 7 controls. For each of the 7 controls individually, this gradient was significantly different from zero: on average, there was a doubling of threshold for every 3° increase in gap size. In contrast, DF's gradient was not significantly different from zero.

To assess whether DF's gradient differed significantly from the population of controls, we used the modified independent samples *t*-test method [35] for comparing the slope of a patient's regression line with those of a sample of controls. For the regression gradients in Figure 5B, just 0.9% of the healthy population are expected to have a gradient lower than DF.

#### DF shows no impairment on an absolute disparity task

On the absolute disparity task (Figure 4Aiii), DF performed as well as the controls. This is a demanding task, and indeed two of the age- and gender-matched controls (F2 and F3) could not perform it at all, at any disparity. We emphasize that this is not because these controls lacked stereo vision, since they had no difficulty with the zero-gap condition, recording thresholds of 0.73 and 0.43 arc min respectively (Supporting Information File S2). Rather, it reflects the well-known difficulty of basing perceptual judgements on absolute disparity alone [1], [2], [37].

We tested whether DF's threshold on the absolute disparity condition differed significantly from those of controls [38], using log-threshold as our performance measure. There was no significant difference, even under the conservative assumption that participants F2 and F3 are assigned the worse of DF's two thresholds, 8.44 arc min (whereas in fact they could not perform this task at all even at 22 arc min)..

**Figure 6:**
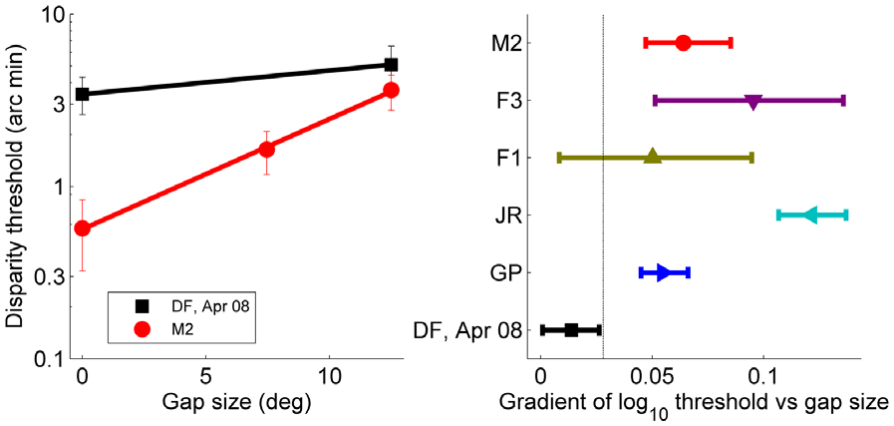
Results of Experiment 1.1S, 160ms stimulus duration. As Figure 5, but for stimulus durations too short to allow eye movements. See Supporting Information File S1 for full data.

In estimating the distance to a target, DF places great weight on binocular vergence information [39]. In theory, she could perform the absolute disparity task by fixating first on the central target and then on the edge of the screen, monitoring the change in vergence. To rule out this strategy, we repeated Experiment 1.1 with a stimulus duration of 160ms, and obtained the same pattern of results (Figure 6). Once again, DF showed similar performance to controls on the absolute-disparity condition; indeed, she performed better than 4/6 controls (DF's threshold 5.1 arc min; Controls GP 2.1, M2 3.6, JR 10.7 and F1, F2, F3 unable to perform the task; see Supporting Information File S2). However, whereas controls found the task substantially easier when the reference surface was introduced, DF once again showed no significant improvement.

In summary, then, DF is not impaired on this very demanding short-duration, absolute-disparity task, despite her extensive damage to visual cortical areas. Rather, she appears to have a specific difficulty in using the relative disparity information provided by the reference surface to improve her sensitivity.

#### Experiment 1.2: perceiving disparity away from the fovea

In Experiment 1.1, the boundary at which relative disparity is available is around the edge of the central target disk, at an eccentricity of 2.8°. Thus, perhaps the simplest explanation for DF's failure to benefit from the relative disparity provided by the reference surface would be that DF can perceive disparity only in the center of her visual field. DF does have a peripheral scotoma beyond 30° eccentricity [25] but this is too far from the fovea to be relevant here.

**Figure 7:**
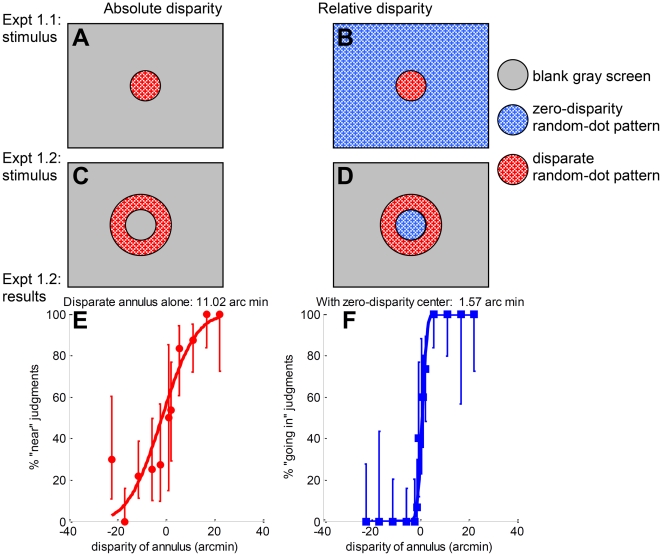
Results of Experiment 1.2, the annulus task. A: “no reference” condition of Expt 1.1; C: Annulus stimulus with blank center. Both these stimuli require an absolute disparity judgment. B: “no gap” condition of Expt 1.1; D: Annulus stimulus when the center is filled with zero-disparity dots. Both these stimuli contain relative disparity information between abutting surfaces. EF: Psychometric functions for patient DF, for the stimuli in C (E) and D (F). The percentage of these judgments are plotted as a function of the disparity of the annulus. Errorbars show 95% confidence intervals based on simple binomial statistics.

In Experiment 1.2, we asked DF to discriminate the depth sign of a annulus of random dots presented for 160ms (Figure 7C) with an inner radius of 2.8°. The center of the annulus was blank gray. DF had some difficulty in learning to perform this demanding short-duration, non-foveal absolute-disparity task, but, as Figure 7E shows, after some practice she was able to perform with a threshold of 11 arc min. This demonstrates that DF does have stereo vision at the eccentricity of the reference surface in Experiment 1.1, ruling out this potential explanation for her poor performance on the no-gap condition.

#### DF shows a weak learning effect with relative disparity

With a repeat of Experiment 1.2 with zero-disparity dots filling the center of the annulus (Figure 7D), DF was not only able to perform the task, but her threshold dropped by an order of magnitude, from 11 arc min to 1.6 arc min. It appears that DF could now benefit from the relative disparity information at this boundary.

**Figure 8:**
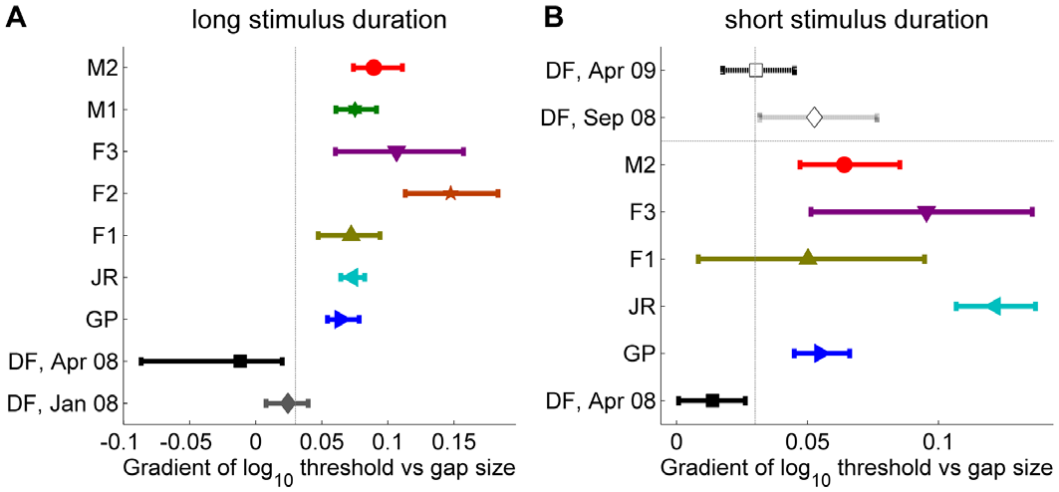
Experiment 1.1, fitted regression gradients from all control subjects and from DF in different testing sessions. AB: Gradients of the regression lines are shown for the different subjects/sessions, for long (A) and short (160ms, B) stimulus durations. DF's data are shown in black/gray. Filled symbols show results from 3 sessions before she was tested on the annulus task (Experiment 1.2, Figure 7, tested in Sep 08); empty symbols show results from 2 sessions carried out after this. Control data are shown with colored symbols. The vertical dashed lines mark the separation between DF's gradients up till April 2008 and those of controls. The horizontal dashed line in **B** separates DF's results after testing with Experiment 1.2 from those obtained before.

We therefore next asked DF to perform the target disk task again, Experiment 1.1S (Figure 4A). DF's performance on the no-reference condition was unchanged, but she now showed an improvement as the reference surface was introduced, bringing her within the same range as controls. Figure 8 summarises all the results obtained on Experiment 1.1 for all subjects and testing sessions, for both long (A) and short (B) durations. The black squares show DF's fitted regression gradients measured in Apr 08; the gray diamond also shows the very similar value obtained in a brief pilot session with DF in Jan 08. The white diamond in Figure 8B show DF's gradient measured in Sep 08, immediately after Experiment 1.2 (see Supporting Information File S2 for the full psychometric functions and fitted thresholds). This gradient falls within the 95% confidence interval of 4/5 controls, putting DF within the normal range for controls, in contrast to the results obtained previously.

This training effect did not persist completely. In Apr 09, DF performed Experiment 1.1S for a fifth time, with the results shown by the white square in Figure 8B (see also Supporting Information File S2). It is difficult to say with confidence whether the effect of the training in Sep 08 persisted unchanged Our best estimate is that the training continued to have some effect, but that it had declined over the intervening six months. However, as we shall demonstrate, this training effect was specific to the exact configuration on which training was carried out.

#### Experiment 1.3: top/bottom surfaces

**Figure 9:**
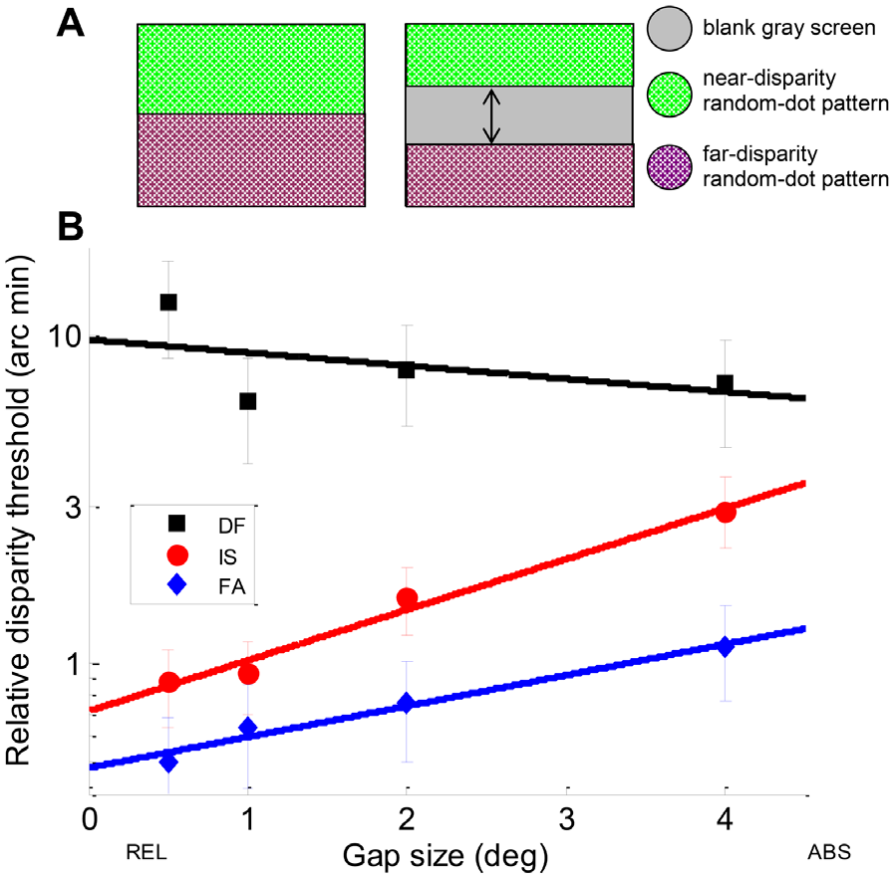
Experiment 1.3: top/bottom closer. Controls perform worse as gap size increases, whereas DF is unaffected. **A**: sketch of stimulus geometry. The top and bottom surfaces always had equal and opposite disparity relative to the fixation point; the task is to say which is closer. We varied the relative disparity between the top and bottom surfaces to obtain the relative disparity threshold for 4 different sizes of gap separating the two surfaces. The “surfaces” were depicted with black and white random dots on a uniform gray background; the gap region was the same uniform gray. **B**: Thresholds as a function of gap size for patient DF (black squares) and two controls (red dots, blue diamonds). See Supporting Information File S3 for full data.

To assess whether the improvement apparently produced by Experiment 1.2 would generalize to a different arrangement of surfaces, in March 2009 we tested DF on the stimulus depicted in Figure 9A. Here, random dots depicted a near surface on the top half of the screen and a far surface on the bottom half (or vice versa). The task was to say whether the top or bottom surface was nearer. The surfaces either directly abutted on another, or were separated by a blank region. In Figure 9B, DF's performance is independent of the size of the gap between the surfaces (gradient not significantly different from zero), whereas control subjects became much better as the gap size reduced (individual gradients significantly positive for both control subjects, bootstrap resampling; full data provided in Supporting Information File S3). Thus, despite the ephemeral training effects on DF's ability to use the relative disparity between adjacent surfaces, she has a significant impairment compared to the ease with which control subjects effortlessly, automatically exploit this information.

### Experiment 2: Sensitivity to slant defined by disparity

**Figure 10:**
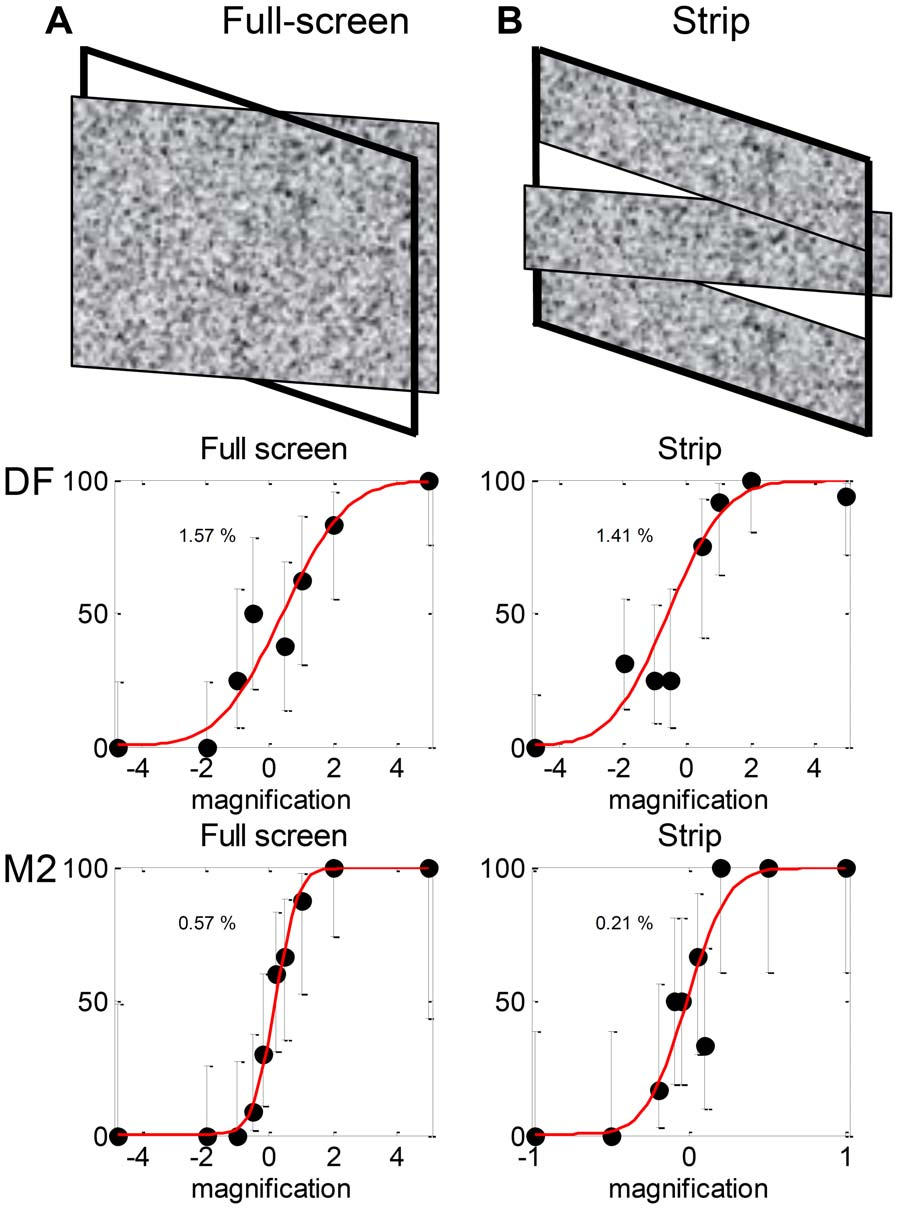
Experiment 2: Disparity-defined slant. A random-dot pattern is magnified horizontally in one eye so as to depict a surface slanted about a vertical axis. **A**: Experiment 2.1, whole display is slanted. **B**: Experiment 2.2, only central strip is slanted. **C**: Psychometric functions and fitted thresholds for patient DF (test session April 2009) and age-matched control M2, for stimulus duration 160ms. Note different axis range for M2, strip condition. See Supporting Information File S3 for full data.

In Experiment 2, we examined DF's sensitivity to changes in disparity across a surface (Figure 1B). Subjects were asked to discriminate the sign of slant defined by binocular disparity, i.e. to report which side of a slanted surface appeared closer. We again compared stimuli with and without relative disparity at surface boundaries. On the full-screen task (Experiment 2.1, Figure 10A), the whole screen depicted a slanted surface, as if the surface of the monitor had been rotated about a vertical axis. On the strip task (Experiment 2.2, Figure 10B), the slanted region was confined to a horizontal strip running across the whole screen between ±4.8° from fixation; the strips at the top and bottom of the screen had zero disparity. Thus, on the full-screen task, adjacent-surface relative-disparity information was available only at the edges of the screen, at least 9.5° from the fixation cross. At such large eccentricities, this information was of little benefit unless the stimulus was displayed for long enough to enable observers to make a saccade to the edge of the monitor. For stimulus presentations of 160ms, the full-screen stimulus effectively contained no adjacent-surface relative disparity information. The strip task, on the other hand, contained adjacent-surface relative-disparity information within 4.8° of the fovea. Many studies have shown that slant perception in control observers is greatly enhanced by such surface boundaries [40], [41].

**Figure 11:**
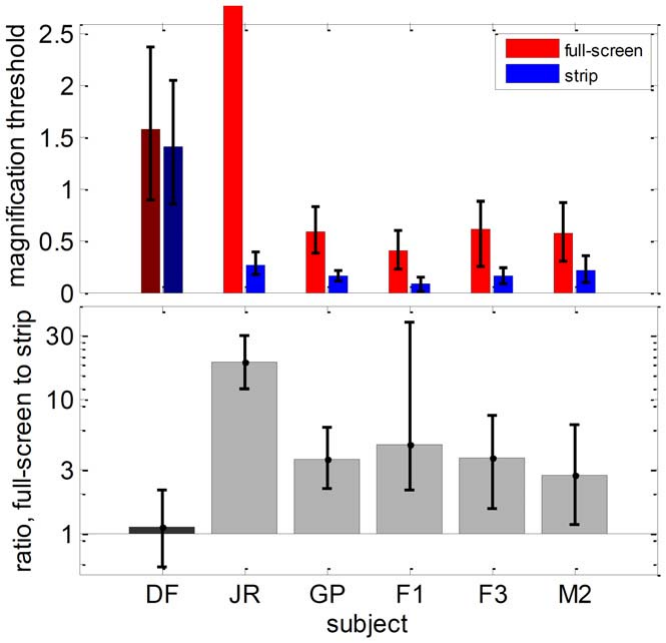
Results of Experiment 2: Disparity-defined slant. **A**: Magnification thresholds for DF and 5 controls on Experiment 2, in the full-screen condition (red, 2.1, Figure 7A) and in the strip condition (blue, 2.2, Figure 7B), stimulus duration 160ms. Subject JR's threshold on the full-screen task was 5%. **B**: Ratio of magnification threshold obtained in the full-screen task to threshold obtained in the strip task. In both cases, error-bars show 95% confidence intervals. See Supporting Information File S3 for full data.

Figure 11A compares thresholds recorded on the two tasks: full-screen (red bars) and strip (blue), both for stimulus durations of 160ms. On the full-screen task, DF's threshold is worse than most control observers', though better than author JR's. But the control subjects all show substantial significant improvements in the strip condition, whereas DF did not. This is quantified in Figure 11B, which plots the ratio of thresholds on the full-screen condition to that on the strip condition. For each of the 5 control observers tested at 160ms, the threshold on the full-screen task was five times greater than that on the strip task (geometric mean). In contrast, DF's thresholds on the two tasks did not differ significantly from each other. This is further evidence that DF is impaired in her ability to benefit from relative disparity information at surface boundaries.

### Experiment 3: Dynamic depth stimuli

#### Experiment 3.1: The rotating-cylinder stimulus

**Figure 12:**
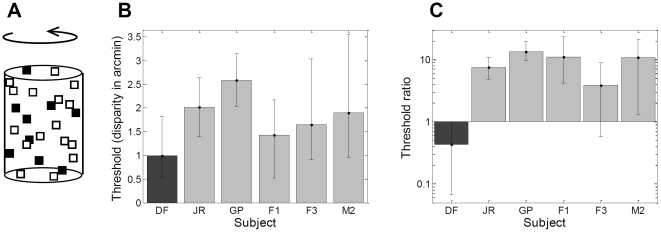
Experiment 3.1: Rotating cylinder. **A**: sketch of the stimulus. Black and white squares move sinusoidally back and forth across a gray background, giving the impression of a glass cylinder covered in dots rotating about its own axis. The cylinder was 5.8° wide×9.2° high. The cylinder completed one rotation every 5s, corresponding to an average dot velocity of 2.6°/s. **B**: Disparity thresholds for DF and 5 controls, i.e. the relative disparity between the front and back surfaces of the cylinder at which performance is 84% correct. **C**: Disparity thresholds on the rotating cylinder task expressed as a fraction of smallest threshold obtained on any front/back discrimination task. See Supporting Information File S3 for full data.

In Experiment 3, we examined DF's ability to use a different form of relative disparity, that between transparently-viewed surfaces (Figure 1D). First we used the “transparent rotating cylinder” task (Experiment 3.1, Figure 12A). Here, dots moving left and right with a sinusoidal velocity profile depict a transparent rotating cylinder, and subjects are asked whether the front surface is moving left or right. This stimulus has two advantages: first, normal human subjects have very low thresholds when using binocular disparity to discriminate front and back surfaces; second, the neurophysiological evidence points strongly to dorsal stream involvement in this task [17], [42]–[45], so one might expect DF to be less impaired here than in Experiment 1.

Indeed, DF could easily perform this task, discriminating front and back surfaces on the basis of disparities of just 1 minute of arc. This is better than any of our controls (Figure 12B), although the large confidence intervals mean we cannot be sure that this difference is significant. DF's threshold on the rotating cylinder task is also twice as good as her performance on any front/back discrimination task in Experiment 1 (Figure 12C), whereas all control subjects were between 3 and 10 times *worse* on the rotating cylinder task. The rotating cylinder stimulus elicits strong activity in dorsal cortical area MT. Potentially, therefore, DF's 20 years of relying on dorsal areas for visual processing may mean she has learnt to use this information more efficiently than control observers.

#### Experiment 3.2: Relative disparity between transparent surfaces

**Figure 13:**
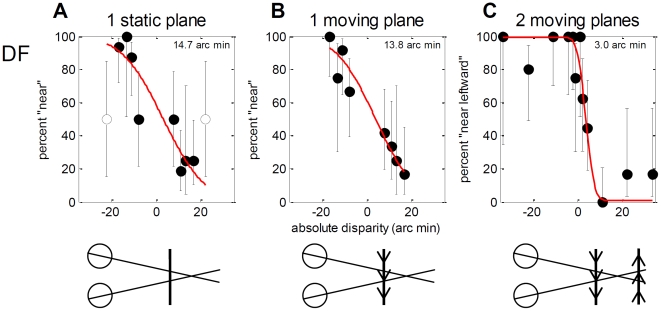
Experiment 3.2: front/back discrimination with static vs moving dots. The stimulus was a patch of light and dark dots 4.5° wide×2.2° high on a gray background. In (A), the dots were static and all in the same depth plane, with absolute disparity indicated on the horizontal axis. The red line shows a cumulative Gaussian fitted to the black dots (white dots represent data not used for fitting); the number in the upper right is the fitted threshold, i.e. the standard deviation of the fitted Gaussian. (B) as (A), except the dots were moving either left or right, with constant speed 2.7°/s. In (A) and (B), the task was to report whether the plane appeared in front of or behind the screen. (C) as (B), except there was a second plane of dots with the opposite direction of motion, and opposite disparity. The two planes of dots moved transparently past one another; the total dot density was thus twice as great in (C) as in (A) and (B). The task in (C) was to report the direction of motion of the front plane. For comparison with A and B, the horizontal axis in C, and the threshold, shows the absolute disparity of the leftward-moving plane. The relative disparity between the two planes was twice this.

**Figure 14:**
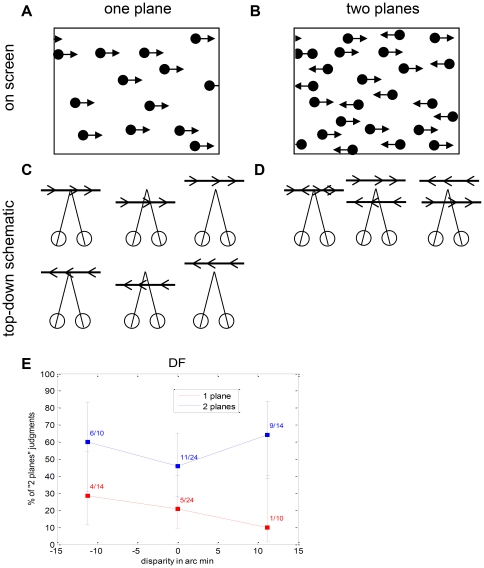
Experiment 3.3: One (A/C) vs two (B/D) planes, defined by moving dots. Each plane was defined by dots moving with the same speed and direction, either left or right. Each plane had a disparity of either 0 or ±11.2 arc min; in the two-plane stimulus with non-zero disparity, the planes had opposite disparity (i.e. the relative disparity between the two planes was either 0 or 22.4 arc min. A/B shows a schematic of an example stimulus on the screen; C/D shows a top-down view of the simulated planes, for each possible stimulus configuration of each type. The number of dots on each plane was constant, i.e. the total number of dots visible was twice as great in the two-plane stimulus (B). The task was to discriminate the one-plane stimuli (A/C) from the two-plane (B/D); this could be done either by detecting the number of directions of motion present (one vs two), or using the density cue.

DF's good performance on Experiment 3.1 might reflect a generally better ability to use absolute disparity in moving stimuli, rather than a genuine ability to use the relative disparity between transparent moving surfaces. To address this, we compared DF's absolute disparity thresholds for an isolated random-dot plane when the dots were static (Figure 13A) versus when they had a constant velocity of 2.7° either leftwards or rightwards (Figure 13B). DF's threshold was the same whether the dots were static or moving. However, her threshold improved greatly when we added a second, transparent plane of dots with opposite disparity and direction of motion (Figure 13C; Figure 14B). The fact that DF was able to benefit from the relative disparity provided by this second plane suggests that it was the relative disparity, not the motion *per se*, which enabled her excellent performance on the rotating cylinder task. That is, DF can instantly and reliably use relative disparity information between moving transparent surfaces, in sharp contrast to the difficulty she experiences in learning to use the relative disparity between spatially separate surfaces.

#### Experiment 3.3: Perceiving transparent surfaces

Curiously, despite this good performance, it transpired that DF in fact only ever perceived one direction of motion when presented with two transparent planes of dots moving in opposite directions. When asked to discriminate such stimuli from a single plane where all the dots moved with the same velocity (Figure 14), she was able to guess slightly above chance (64 out of 96, *p*<0.001) after practice, while but she always reported phenomenal percepts of single-direction motion. For any normally-sighted individual, the difference between one or two planes is obvious: one immediately perceives either one or two directions of motion. Evidently DF could still use information about transparent planes defined by motion and/or disparity to improve her depth discrimination thresholds – as well as or better than any of the control subjects we examined – despite being unable to perceive this information consciously.

### Anti-correlated random-dot patterns

We tested DF on anti-correlated versions of the target-disk stimulus of Experiment 1.1 (Figure 4Ai), and the rotating cylinder of Experiment 3.1 (Figure 12A), using disparities with which she had performed near-perfectly for correlated stimuli. In both cases, she performed at chance, just like control observers. Specifically, on Experiment 1.1L with disparity ±22 arc min, DF answered correctly on 59/60 trials when the stimulus was correlated, and on 20/40 trials when it was anti-correlated. On Experiment 3.1 with a relative disparity of 45 arc min between the front and back surfaces of the cylinder, DF answered correctly on 32/32 trials when the stimulus was correlated, and on 8/16 trials when it was anti-correlated. Thus, neither DF nor controls can perceptually discriminate the sign of depth in anti-correlated stimuli, despite the fact that activity in many cortical areas contains the necessary information [14], [17].

## Discussion

### DF performs normally on many stereo tasks

Given the extensive damage to several areas of DF's visual cortex, perhaps the most surprising finding of the present paper is how little she is impaired on stereoscopic vision. Previous investigations have established that DF has functional stereo vision [25], [46], although she was reported to have poorer stereoacuity than controls. Our results show that DF's poorer stereoacuity in the earlier studies reflected the task (a standard clinical test of the relative disparity between two surfaces), rather than a general stereo deficit. On discriminating depth in isolated stimuli, or judging the relative depth between transparent planes, DF performs as well as controls. For example, on Experiment 1.1, 3 out of 5 age-matched controls with normal stereo vision could not discriminate the depth sign of a briefly-presented isolated target for any disparity, while author JR, an experienced stereo observer twenty years younger than DF, could not match her threshold. On the rotating cylinder task, DF outperformed all of the controls we examined. Thus, the particular impairments that we have been able to record certainly do not reflect a general loss of stereoacuity or a difficulty in achieving stereoscopic fusion.

### DF is impaired in using relative disparity between different visual-field locations

The major impairment is DF's ability to exploit relative disparity between nearby surfaces. Thus, in Experiments 1.1 and 1.3, DF's performance drops dramatically below that of controls when the comparison surfaces are close together, because controls are able to judge the relative disparity between surfaces more accurately than the absolute disparity of either one alone. In the disparity-defined slant task, the performance of controls improves when the slant is confined to a central strip (Experiment 2.2S), because they benefit from the relative-disparity information available at the strip's boundary. In contrast, DF shows no such improvement. Thus, three different stimulus geometries all indicate that DF has difficulties in using relative disparity at surface boundaries.

The results of Experiment 1 are consistent with previous suggestions that the dorsal stream predominantly handles “coarse” stereopsis, e.g. absolute disparity signals representing objects' locations in 3D space, whereas the ventral stream handles “fine” stereopsis, including relative disparity between adjacent surfaces [2], [47], [48].

The firing of dorsal stream neurons contains information about fine disparities in the visual scene, but whether this information contributes to perception depends critically both on the stimulus configuration and on training [44], [45], [47]–[49]. It is possible that, over the 20 years since her lesion, DF has learnt to make particularly good use of the fine-disparity information available in V5/MT and other dorsal areas. Since the rotating cylinder stimulus is especially effective in stimulating V5/MT neurons [42], a task that demands use of this information may actually benefit DF.

### Possible interpretations

Given that DF's major cortical lesion is to the lateral occipital area LO, we suggest that this brain area may be critical for achieving the ultra-fine sensitivity that control subjects automatically display on these tasks. DF's spared ability to use the relative disparity between transparent surfaces in this way is consistent with imaging and neurophysiological evidence suggesting that this ability depends upon dorsal areas such as MT [42], [50], [51], perhaps because such disparity/motion parallax information can be important for guiding movements. There is in fact independent evidence that DF can exploit motion parallax, as well as stereo information, when reaching to grasp slanted objects [52], [53].

This then raises the question of how precisely area LO enables controls to exploit relative disparity information at surface boundaries. Human imaging studies suggest that LO is involved in the processing of stereo-defined shape, including stereo-defined boundaries [54]–[57]. However, DF's ability to use relative disparity information after appropriate training suggests that she has not simply lost the neuronal tissue which computes relative disparity at surface boundaries, nor the ability to exploit these signals in principle. Rather, it may be that her lesion makes it difficult for her to identify the appropriate neuronal signals for a given relative disparity task. For example, DF may have difficulty identifying disparity boundaries between surfaces. Her good performance on transparent stimuli may reflect the fact that these offer the same relative disparity information widely throughout the stimulus.

One possibility to consider is that DF's underlying deficit is in perceiving, or attending to, both surfaces simultaneously. DF's subjective reports certainly indicate that, while aware of performing more accurately in trial blocks when gap size was smaller, she had no explicit perceptual awareness that the reference surround had moved closer. But it has been apparent for many years that DF's perception and behaviour can be strongly influenced by stimuli that she cannot consciously discriminate [25], [27], [58]. Indeed we have internal evidence of this within the present study, in that DF was surprisingly unable to perceive superimposed transparent planes defined by motion and/or disparity – she invariably saw motion in only one direction. Yet she was still able to use the relative disparity between two such planes to improve her depth discrimination thresholds – as well as or better than our control subjects. This demonstrates that DF's difficulty in using relative disparity information between adjacent surfaces is logically distinct from any difficulty she may have in perceiving or attending to both surfaces. In any case, there is also clear independent evidence from other contexts that DF can combine visual information from the peripheral visual field with central (target) information quite efficiently, for example in order to avoid obstacles during reaching [59].

### Conclusions

We have conducted a detailed, quantitative examination of stereo vision in patient DF, who has visual agnosia following cortical damage to predominantly ventral areas. On many stereo tasks, DF performs as well as controls. However, our tests reveal a very specific difficulty, namely in the use of relative disparity between spatially separate locations (Figure 1C). This finding suggests that ventral stream areas may usually be key in achieving the enhanced stereo vision that characterizes normal performance on such tasks.

## Supporting Information

File S1 Note on fitting psychometric functions.
(0.20 MB PDF)

File S2 Psychophysics data from Expt 1.1.
(0.45 MB PDF)

File S3 Psychophysics data from Expts 1.3, 2 and 3.1.
(0.32 MB PDF)

## References

[1] Westheimer G (1979). Cooperative neural processes involved in stereoscopic acuity.. Exp Brain Res.

[2] Parker AJ (2007). Binocular depth perception and the cerebral cortex.. Nat Rev Neurosci.

[3] Thomas OM, Cumming BG, Parker AJ (2002). A specialization for relative disparity in V2.. Nat Neurosci.

[4] Bredfeldt CE, Cumming BG (2006). A simple account of cyclopean edge responses in macaque V2.. J Neurosci.

[5] von der Heydt R, Zhou H, Friedman HS (2000). Representation of stereoscopic edges in monkey visual cortex.. Vision Res.

[6] Umeda K, Tanabe S, Fujita I (2007). Representation of stereoscopic depth based on relative disparity in macaque area V4.. J Neurophysiol.

[7] Janssen P, Vogels R, Orban GA (1999). Macaque inferior temporal neurons are selective for disparity-defined three-dimensional shapes.. Proc Natl Acad Sci U S A.

[8] Janssen P, Vogels R, Orban GA (2000). Three-dimensional shape coding in inferior temporal cortex.. Neuron.

[9] Tsao DY, Vanduffel W, Sasaki Y, Fize D, Knutsen TA (2003). Stereopsis activates V3A and caudal intraparietal areas in macaques and humans.. Neuron.

[10] Neri P, Bridge H, Heeger DJ (2004). Stereoscopic processing of absolute and relative disparity in human visual cortex.. J Neurophysiol.

[11] Haefner RM, Cumming BG (2008). Adaptation to natural binocular disparities in primate V1 explained by a generalized energy model.. Neuron.

[12] Cogan AI, Lomakin AJ, Rossi AF (1993). Depth in anticorrelated stereograms: effects of spatial density and interocular delay.. Vision Res.

[13] Cumming BG, Shapiro SE, Parker A (1998). Disparity detection in anticorrelated stereograms.. Perception.

[14] Cumming BG, Parker AJ (1997). Responses of primary visual cortical neurons to binocular disparity without depth perception.. Nature.

[15] Read JCA, Eagle RA (2000). Reversed stereo depth and motion direction with anti-correlated stimuli.. Vision Res.

[16] Ohzawa I, DeAngelis GC, Freeman RD (1990). Stereoscopic depth discrimination in the visual cortex: neurons ideally suited as disparity detectors.. Science.

[17] Krug K, Cumming BG, Parker AJ (2004). Comparing perceptual signals of single V5/MT neurons in two binocular depth tasks.. J Neurophysiol.

[18] Neri P, Parker AJ, Blakemore C (1999). Probing the human stereoscopic system with reverse correlation.. Nature.

[19] Masson GS, Busettini C, Miles FA (1997). Vergence eye movements in response to binocular disparity without depth perception.. Nature.

[20] Takemura A, Inoue Y, Kawano K, Quaia C, Miles FA (2001). Single-unit activity in cortical area MST associated with disparity-vergence eye movements: evidence for population coding.. J Neurophysiol.

[21] Tanabe S, Umeda K, Fujita I (2004). Rejection of false matches for binocular correspondence in macaque visual cortical area V4.. J Neurosci.

[22] Janssen P, Vogels R, Liu Y, Orban GA (2003). At least at the level of inferior temporal cortex, the stereo correspondence problem is solved.. Neuron.

[23] Bridge H, Parker AJ (2007). Topographical representation of binocular depth in the human visual cortex using fMRI.. J Vis.

[24] Preston TJ, Li S, Kourtzi Z, Welchman AE (2008). Multivoxel pattern selectivity for perceptually relevant binocular disparities in the human brain.. J Neurosci.

[25] Milner AD, Perrett DI, Johnston RS, Benson PJ, Jordan TR (1991). Perception and action in ‘visual form agnosia’.. Brain.

[26] James TW, Culham J, Humphrey GK, Milner AD, Goodale MA (2003). Ventral occipital lesions impair object recognition but not object-directed grasping: an fMRI study.. Brain.

[27] Goodale MA, Milner AD, Jakobson LS, Carey DP (1991). A neurological dissociation between perceiving objects and grasping them.. Nature.

[28] Brainard DH (1997). The Psychophysics Toolbox.. Spat Vis.

[29] Pelli DG (1997). The VideoToolbox software for visual psychophysics: transforming numbers into movies.. Spat Vis.

[30] Julesz B (1971). Foundations of cyclopean perception.

[31] Wichmann FA, Hill NJ (2001). The psychometric function: I. Fitting, sampling, and goodness of fit.. Percept Psychophys.

[32] Wilson EB (1927). Probable inference, the law of succession, and statistical inference.. Journal of the American Statistical Association.

[33] Efron B (1979). Bootstrap methods: another look at the jackknife.. Annals of Statistics.

[34] Wichmann FA, Hill NJ (2001). The psychometric function: II. Bootstrap-based confidence intervals and sampling.. Percept Psychophys.

[35] Crawford JR, Garthwaite PH (2004). Statistical methods for single-case studies in neuropsychology: comparing the slope of a patient's regression line with those of a control sample.. Cortex.

[36] Crawford JR, Garthwaite PH (2007). Comparison of a single case to a control or normative sample in neuropsychology: development of a Bayesian approach.. Cogn Neuropsychol.

[37] Erkelens CJ, Collewijn H (1985). Motion perception during dichoptic viewing of moving random-dot stereograms.. Vision Res.

[38] Crawford JR, Garthwaite PH (2002). Investigation of the single case in neuropsychology: confidence limits on the abnormality of test scores and test score differences.. Neuropsychologia.

[39] Mon-Williams M, Tresilian JR, McIntosh RD, Milner AD (2001). Monocular and binocular distance cues: insights from visual form agnosia I (of III).. Exp Brain Res.

[40] Gillam B, Flagg T, Finlay D (1984). Evidence for disparity change as the primary stimulus for stereoscopic processing.. Percept Psychophys.

[41] Gillam B, Blackburn S, Brooks K (2007). Hinge versus twist: the effects of ‘reference surfaces’ and discontinuities on stereoscopic slant perception.. Perception.

[42] Bradley DC, Chang GC, Andersen RA (1998). Encoding of three-dimensional structure-from-motion by primate area MT neurons.. Nature.

[43] DeAngelis GC, Cumming BG, Newsome WT (1998). Cortical area MT and the perception of stereoscopic depth.. Nature.

[44] Dodd JV, Krug K, Cumming BG, Parker AJ (2001). Perceptually bistable three-dimensional figures evoke high choice probabilities in cortical area MT.. J Neurosci.

[45] Uka T, DeAngelis GC (2004). Contribution of area MT to stereoscopic depth perception: choice-related response modulations reflect task strategy.. Neuron.

[46] Marotta JJ, Behrmann M, Goodale MA (1997). The removal of binocular cues disrupts the calibration of grasping in patients with visual form agnosia.. Exp Brain Res.

[47] Uka T, DeAngelis GC (2006). Linking neural representation to function in stereoscopic depth perception: roles of the middle temporal area in coarse versus fine disparity discrimination.. J Neurosci.

[48] Roe AW, Parker AJ, Born RT, DeAngelis GC (2007). Disparity channels in early vision.. J Neurosci.

[49] Chowdhury SA, DeAngelis GC (2008). Fine discrimination training alters the causal contribution of macaque area MT to depth perception.. Neuron.

[50] Bradley DC, Qian N, Andersen RA (1995). Integration of motion and stereopsis in middle temporal cortical area of macaques.. Nature.

[51] Spang K, Morgan M (2008). Cortical correlates of stereoscopic depth produced by temporal delay.. J Vis.

[52] Dijkerman HC, Milner AD, Carey DP (1996). The perception and prehension of objects oriented in the depth plane. I. Effects of visual form agnosia.. Exp Brain Res.

[53] Dijkerman HC, Milner AD, Carey DP (1999). Motion parallax enables depth processing for action in a visual form agnosic when binocular vision is unavailable.. Neuropsychologia.

[54] Gilaie-Dotan S, Ullman S, Kushnir T, Malach R (2002). Shape-selective stereo processing in human object-related visual areas.. Hum Brain Mapp.

[55] Mendola JD, Dale AM, Fischl B, Liu AK, Tootell RB (1999). The representation of illusory and real contours in human cortical visual areas revealed by functional magnetic resonance imaging.. J Neurosci.

[56] Kourtzi Z, Kanwisher N (2001). Representation of perceived object shape by the human lateral occipital complex.. Science.

[57] Grill-Spector K, Kourtzi Z, Kanwisher N (2001). The lateral occipital complex and its role in object recognition.. Vision Res.

[58] Humphrey GK, Goodale MA, Gurnsey R (1991). Orientation Discrimination in a Visual Form Agnosic: Evidence from the McCollough Effect Psychological Science.

[59] Rice NJ, McIntosh RD, Schindler I, Mon-Williams M, Demonet JF (2006). Intact automatic avoidance of obstacles in patients with visual form agnosia.. Exp Brain Res.

