# Supplementary material for: Stereoscopic Vision in the Absence of the Lateral Occipital Cortex
Source: PLoS One. 2010 Sep 7;5(9):e12608. doi: 10.1371/journal.pone.0012608 (PMC2935377; doi:10.1371/journal.pone.0012608)
Supplement: File S1 — Note on fitting psychometric functions. (0.20 MB PDF) [file pone.0012608.s001.pdf]

Fitting psychometric functions

Psychometric functions were fitted with a cumulative Gaussian allowing for a lapse rate *l* (Equation 1):

$$p(x;\mu,\sigma) = \frac{1}{2} + \frac{(1-l)}{2} erf\left(\frac{x-\mu}{\sigma\sqrt{2}}\right)$$

Where *x* is disparity, *p* is the probability of making a "far" response. μ represents the point of subjective equivalence, where the subject is equally likely to respond "near" or "far", and σ is the threshold: the amount of positive disparity required for the subject to respond "far" 82% of the time. Data consisted of a set of n_i responses at disparity x_i,m_i of which were "far". Assuming simple binomial statistics, the probability of recording m_i "far" responses when the probability of a far response is

$$P_i = \frac{n_i!}{m_i!(n_i - m_i)!}(1-p(x_i;\mu,\sigma))^{n_i - m_i}(p(x_i;\mu,\sigma))^{m_i}$$

We chose μ and σ so as to maximize the total likelihood of the data-set. We did this by maximizing

$$\arg\max_{\mu,\sigma}\{(n_i - m_i)\ln(1-p(x_i;\mu,\sigma)) + m_i \ln p(x_i;\mu,\sigma)\}$$

Supp Equation 1

The lapse rate was included to allow for the fact that subjects, being human, occasionally press the wrong button. For example, consider subject M2 in Supp Fig 2. Suppose that this subject's true threshold is σ=0.2 arcmin. At 5arcmin, 25 times above threshold, an ideal observer would never make any errors. Thus in Equation with no lapse rate, *l*=0, the chance of an error is too small for Matlab to calculate: the logarithm in Supp Equation 1 is -∞, indicating impossibility. Yet in one out of the ten trials we performed at 5 arcmin, this subject lost concentration for a moment and accidentally pressed the wrong button. Because the *l*=0 model does not allow for human error, it is forced to conclude that this button-press indicated M2 could not correctly perceive the stimulus at 5 arcmin. It therefore has to vastly overestimate the threshold σ, meaning that the fit is dominated by this one error and gives a very poor account of the remaining data-points. Clearly, a more realistic model would allow for human fallibility. This is what the lapse rate achieves. For example, with *l*=0.01 as used in our fitting, the log-probability of an error at 5 arcmin is -5.3, compared to –infinity at *l*=0. The fitted threshold is then determined, correctly, by the data-points in the vicinity of zero. The particular value of the lapse rate is not important, so long as it is large enough to make a button-press error within the bounds of possibility, and not so large that subjects would be completely unreliable. Changing *l* only alters the precise level of the horizontal portion of the red line showing the fitted function, so values of *l* between 0.0005 and 0.05 tend to give very similar threshold estimates. For this reason, and to avoid over-fitting, we did not fit *l* individually for each subject, but fixed it at 0.01. See Wichmann & Hill (2001) for detailed discussions and simulations.

Wichmann, F. A. & Hill, N. J. The psychometric function: II. Bootstrap-based confidence intervals and sampling. Percept Psychophys 63, 1314-29 (2001).
