# Supplementary material for: Stereoscopic Vision in the Absence of the Lateral Occipital Cortex
Source: PLoS One. 2010 Sep 7;5(9):e12608. doi: 10.1371/journal.pone.0012608 (PMC2935377; doi:10.1371/journal.pone.0012608)
Supplement: File S2 — Psychophysics data from Expt 1.1. (0.45 MB PDF) [file pone.0012608.s002.pdf]

Experiment 1.1: Front/back discrimination of a target disk

In this section, we show all data collected on this task, for both long and short stimulus presentations. The panels on the left show individual psychometric functions, i.e. percentage of responses in which the central disk was judged to be in front, plotted as a function of its disparity, in minutes of arc (positive = crossed). The size of the gap between target and reference surfaces is given above each panel. Errorbars on each data-point show the 95% confidence intervals, assuming simple binomial statistics. Note that some subjects could not perform the task at the larger gap sizes. This was not due to a restricted range of disparities: for these subjects, we explored a range of disparities up to 22arcmin, with no evidence that performance was improving above chance. Beyond 22 arcmin, disparities are approaching Panum's fusional limit for most subjects.

The red curve shows the fitted cumulative Gaussian function. The threshold (fitted SD) is printed at the top left of each panel. Data-points shown in white, rather than black, were not used for fitting. These are mainly large disparities, evidently beyond the subjects' fusional limit, where performance was returning back towards chance. In the absence of a reference surface, some control subjects never rose above chance; these data-points are also shown in white since no meaningful threshold could be fitted.

The right-hand panel in each row shows the fitted thresholds (red dots) plotted as a function of gap size. The errorbars in this plot are the 95% confidence intervals on the fitted thresholds, estimated by bootstrap resampling. The thick blue line shows fitted regression line, fitting $\log_{10}$(threshold in min) as a linear function of gap size in degrees. The shaded blue region shows the 95% confidence interval for the fitted regression. That is, for each gap-size we calculated the values of 10,000 resampled regression lines at that point, and shaded the 95% confidence interval. This takes into account variation in the intercept as well as gradient, which is why the boundaries of the shaded region are not necessarily straight. The numbers at the bottom of each panel show the gradient of the fitted regression line, and the 2.5% and 97.5% percentiles of the fitted gradient.

All data of Patient DF:

Jan 08: long durations

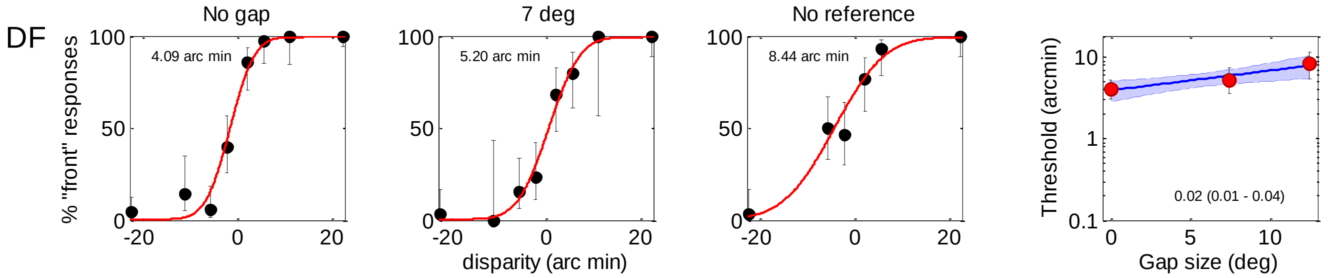

Apr 08: long durations

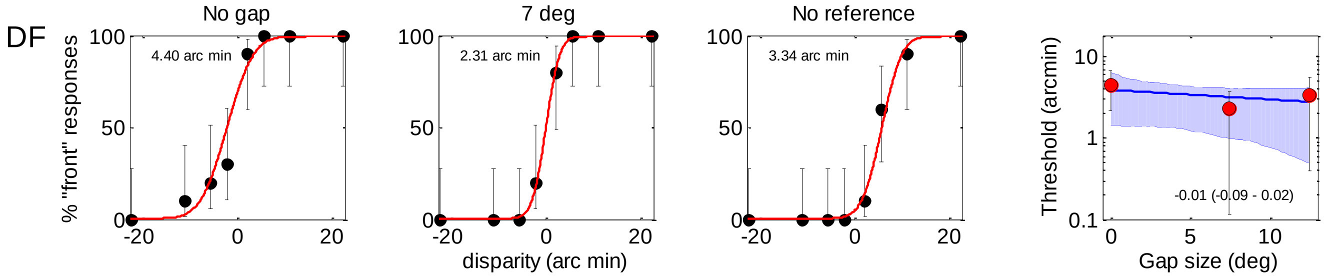

Apr 08: short durations

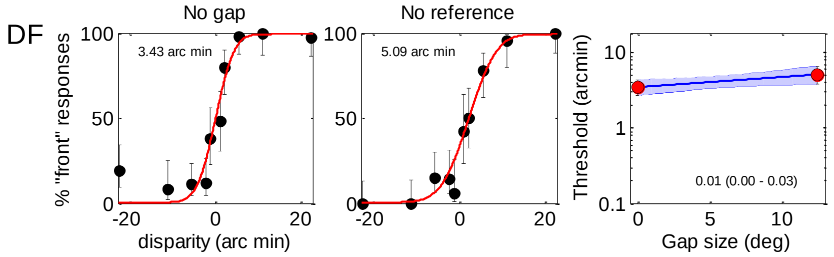

Sep 08: short durations

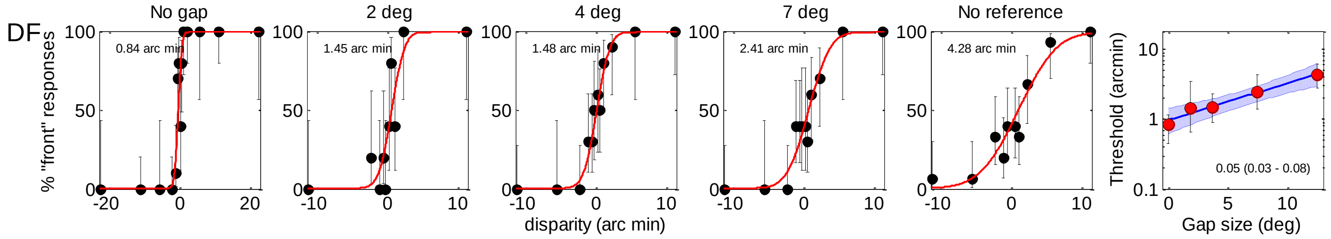

Apr 09: short durations

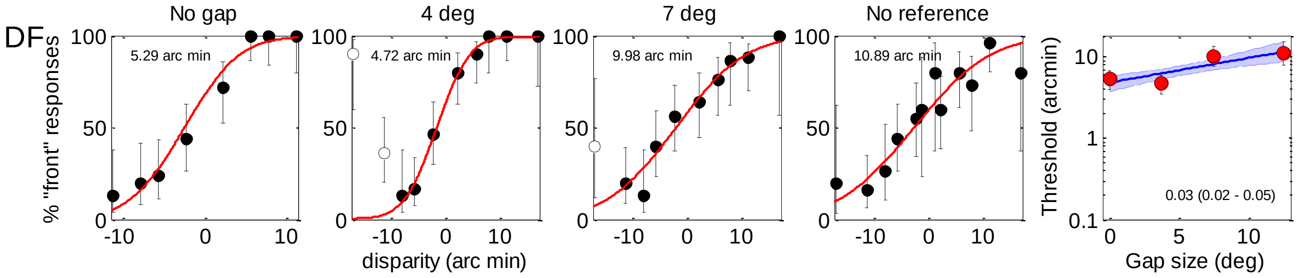

Figure S1. All data collected for Patient DF on the task requiring front/back discrimination of a target disk, on dates and at stimulus durations indicated above each row of panels. See text above for detailed explanation of what is shown in each panel.

Control subjects: long durations

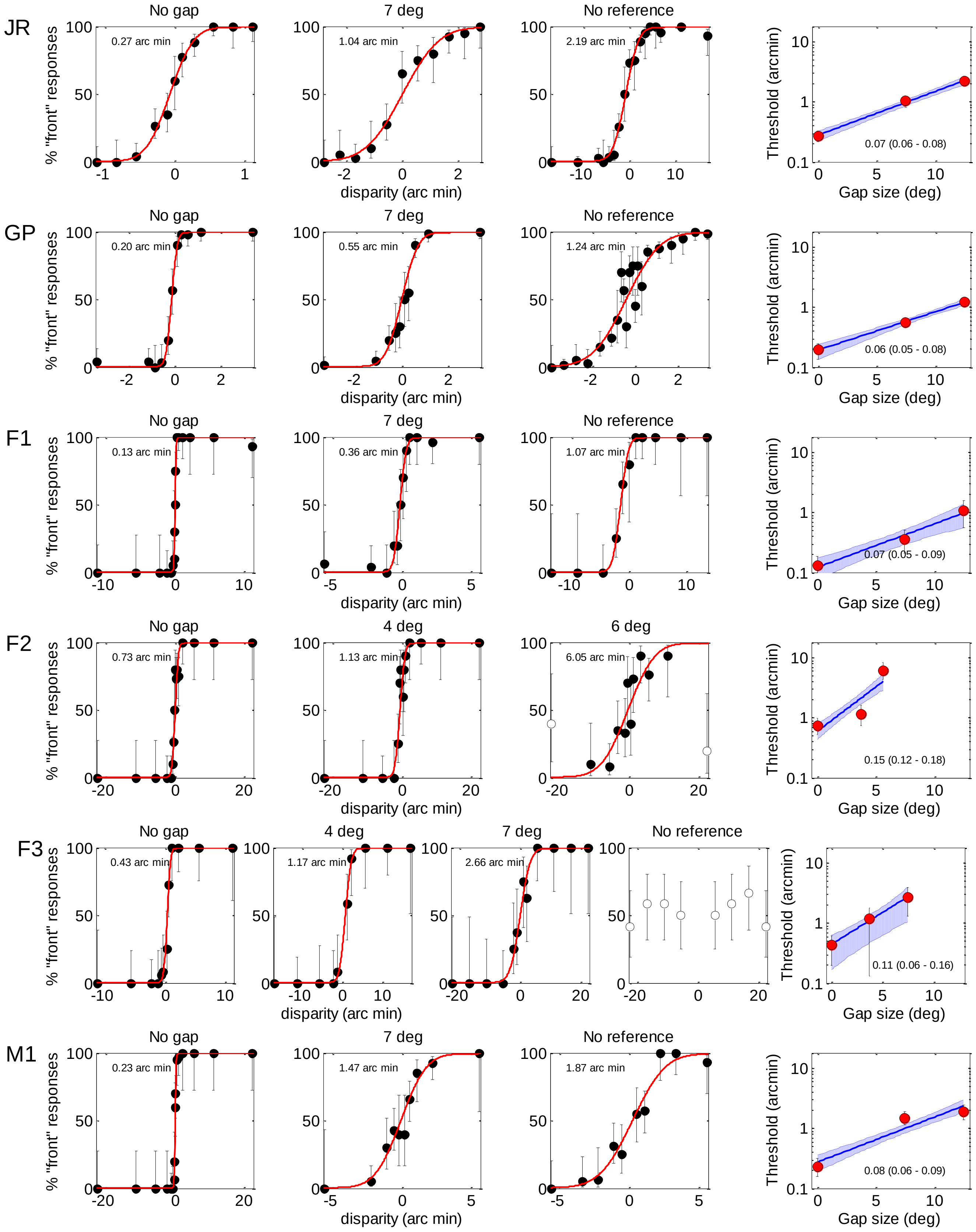

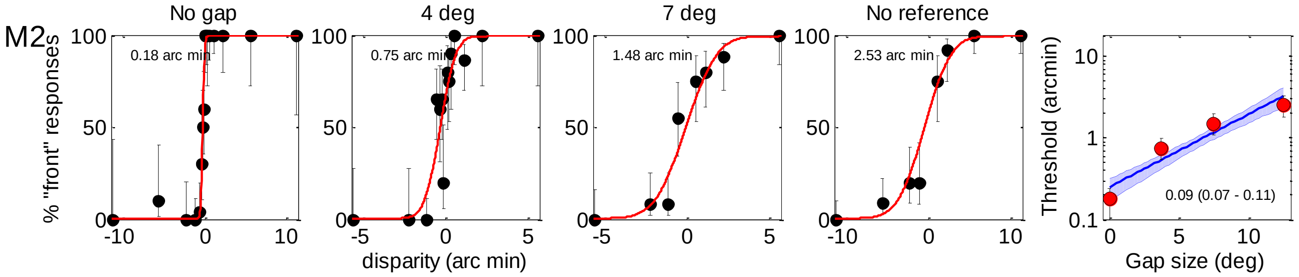

Figure S2. All data collected from control subjects in the long-duration condition of the target-disk task.

Control subjects: short durations (160ms)

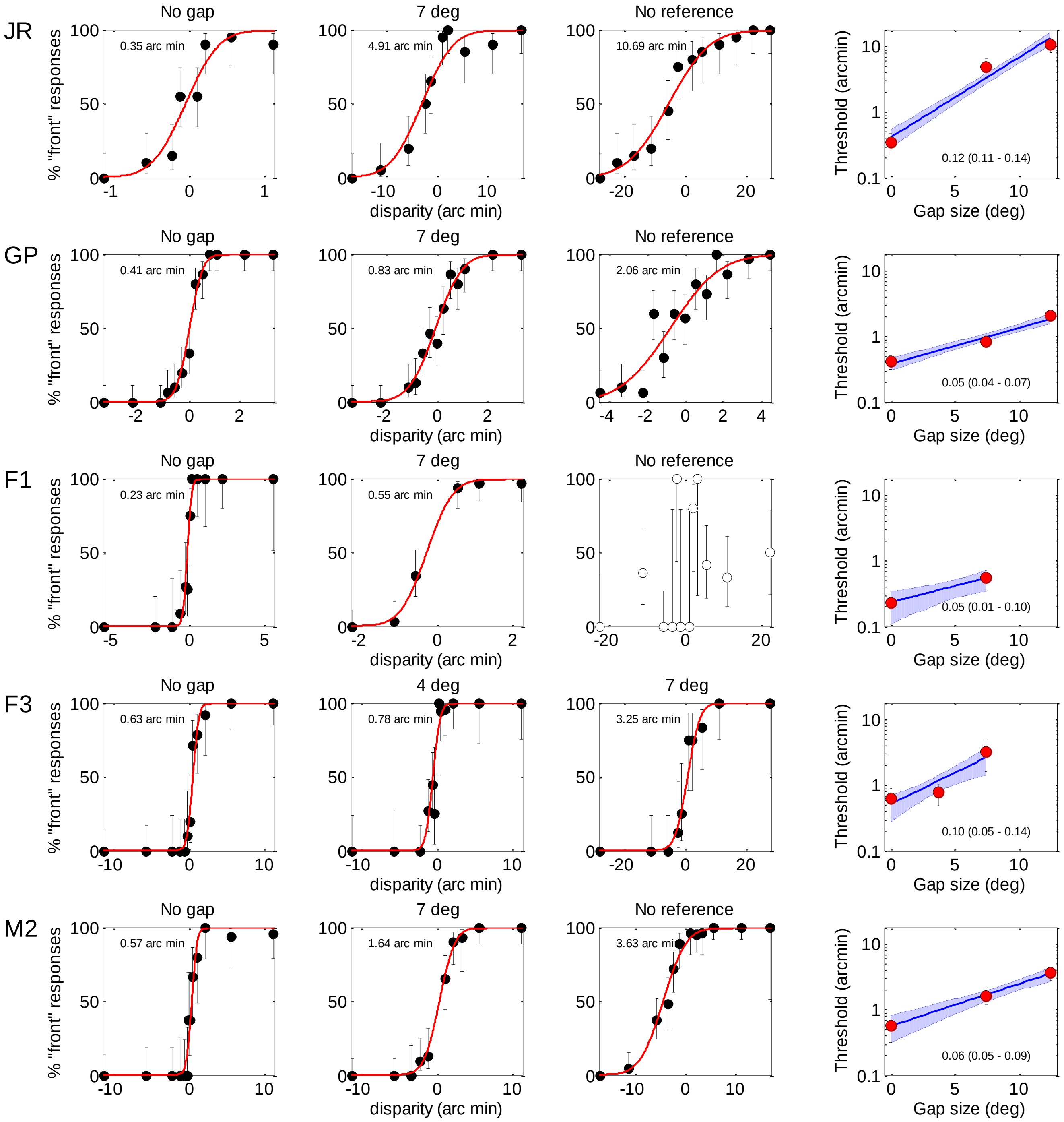

Figure S3. All data collected from control subjects in the short-duration condition of the target-disk task.

Possible monocular cues

One potential problem with introducing a gap around the target whose disparity is to be detected, is that it may introduce a monocular cue to depth. On trials where the target is presented in front of the screen, its left half-image is shifted slightly rightward on the screen, and vice versa. To reduce the salience of this cue, we initially jittered the position of the target randomly on each trial, picking its cyclopean position from a uniform distribution centered on the screen but with a range of 40 pixels, 0.75°, horizontally. For the long-duration experiment, this jitter was present on all trials where a gap existed between target and reference. For the short-duration experiment, our first control subject (M2) reported that he was interpreting this jitter as a spurious depth cue. Because in reality target cyclopean location provided no cue to depth, this resulted in a slight increase in his thresholds. Experiments on M2 and F1 demonstrated that, for both subjects, introducing the jitter slightly increased thresholds. The effect was very slight (Table), but because the jitter was only applied where there was a gap, it would tend to exaggerate the effect of gap size on thresholds. We therefore ceased applying the jitter: the results shown elsewhere for M2 and F1 are for the no-jitter condition, and no jitter was applied for any of the other control subjects in the short-duration experiments. For the sessions in January and April 2008, Patient DF was tested with the jitter applied, in both long- and short-duration conditions . In theory, this could have degraded her performance in the conditions with gap>0. The fact that Patient DF actually performed better on these conditions (both relative to her performance with no gap, and relative to several of the control subjects) thus strengthens the conclusion that she has a specific impairment regarding relative disparity between adjacent surfaces. For DF's sessions in September 2008 and April 2009, no jitter was applied. In practice, it is highly unlikely that DF could have used the jitter cue. The size of the jitter was less than a degree, and DF's visual form agnosia makes it unlikely she would be unable to make such fine discriminations about the precise position of the target. Even for the control subjects, the effect of jitter was tiny, some 10 *seconds* of arc: far too small to explain the results we observe.

Subject	Gap=7.5° No jitter	Gap=7.5° 0.75° jitter	No background No jitter	No background 0.75° jitter
M1	1.50min	1.81min	3.04min	3.42min
F1	0.50min	0.61min		

Table S1. Effect of introducing a jitter into the target location, for 2 control subjects. Entries show fitted SD of psychometric function, for 2 different gap sizes, both with and without jitter, for the short-duration front/back discrimination task. The jitter tends to increase thresholds by around 20%. Subject F1 could not perform the short-duration no-background task with or without a jitter.
