# Supplementary material for: Stereoscopic Vision in the Absence of the Lateral Occipital Cortex
Source: PLoS One. 2010 Sep 7;5(9):e12608. doi: 10.1371/journal.pone.0012608 (PMC2935377; doi:10.1371/journal.pone.0012608)
Supplement: File S3 — Psychophysics data from Expts 1.3, 2 and 3.1. (0.32 MB PDF) [file pone.0012608.s003.pdf]

Experiment 1.3: Top/bottom closer task

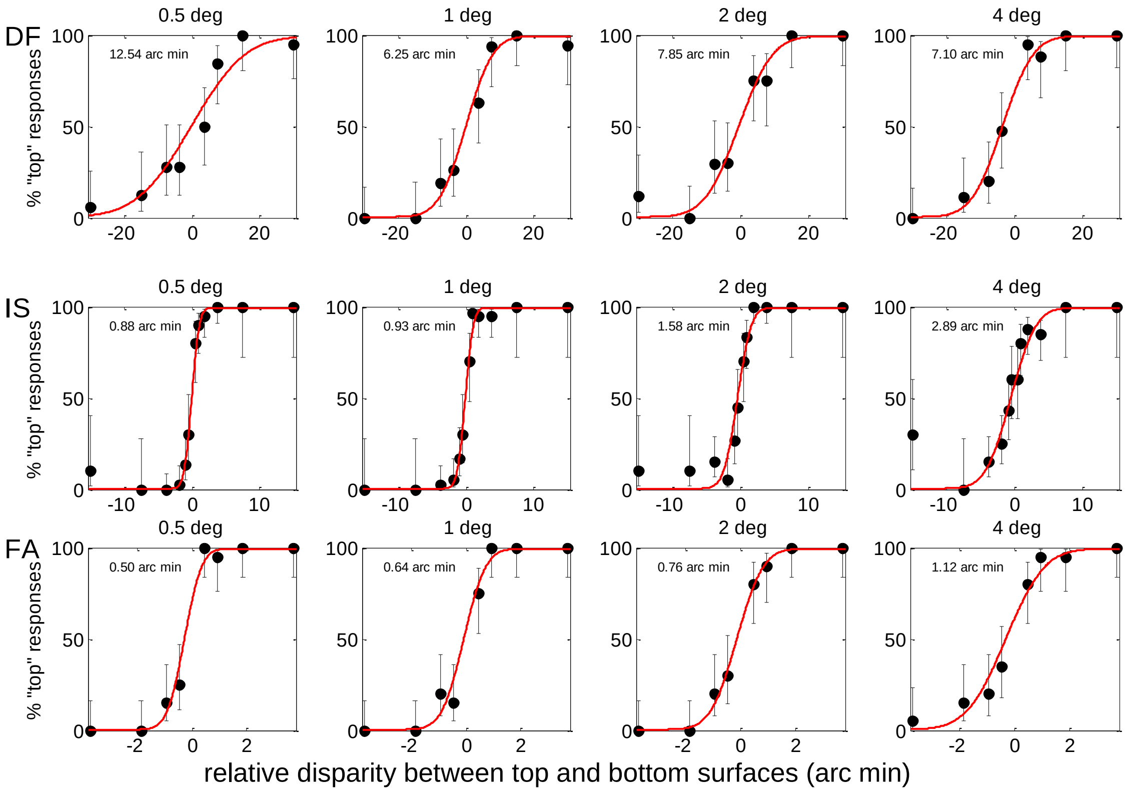

Figure S4. All data collected from DF and 2 control subjects on the task requiring a judgment of whether the top or bottom surface was closer.

Experiment 2: Discrimination of disparity-defined slant

This section shows all individual psychometric functions for the slant discrimination experiment. The horizontal axis shows the percentage horizontal magnification by which one eye's image was expanded relative to the other. The sign of this magnification is taken to be positive if the right eye's image was expanded and the left contracted, and negative otherwise. Left-hand panels show results when magnification was applied only to a horizontal strip across the screen (Figure 3B), and right-hand panels when it was applied to the whole image (Figure 3A).

All data of Patient DF:

Apr 08: long durations

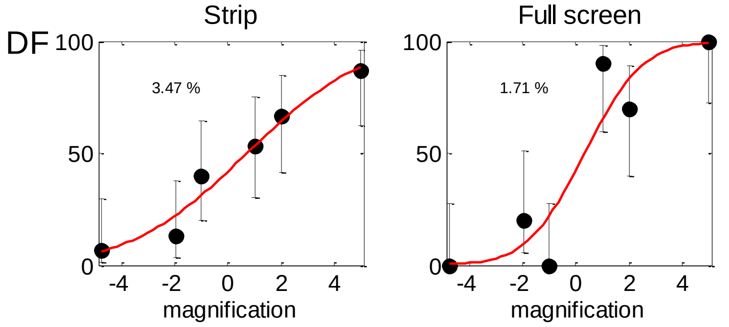

Apr 08: short durations

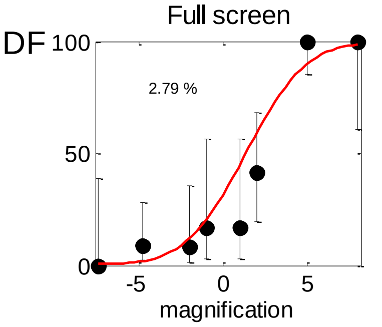

Apr 09: short durations (160ms)

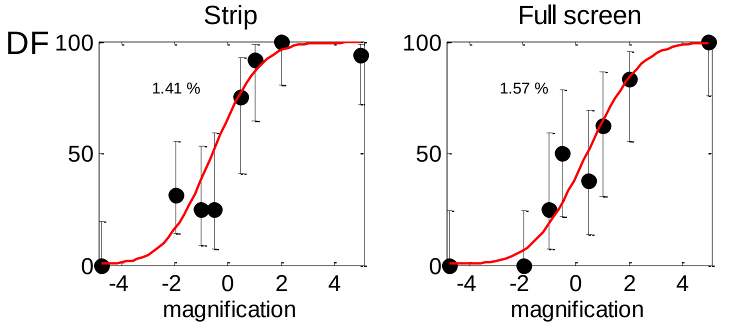

Figure S5. All data collected for Patient DF on the disparity-defined slant discrimination task, on dates and at stimulus durations indicated above each row of panels.

Control subjects: short durations (160ms)

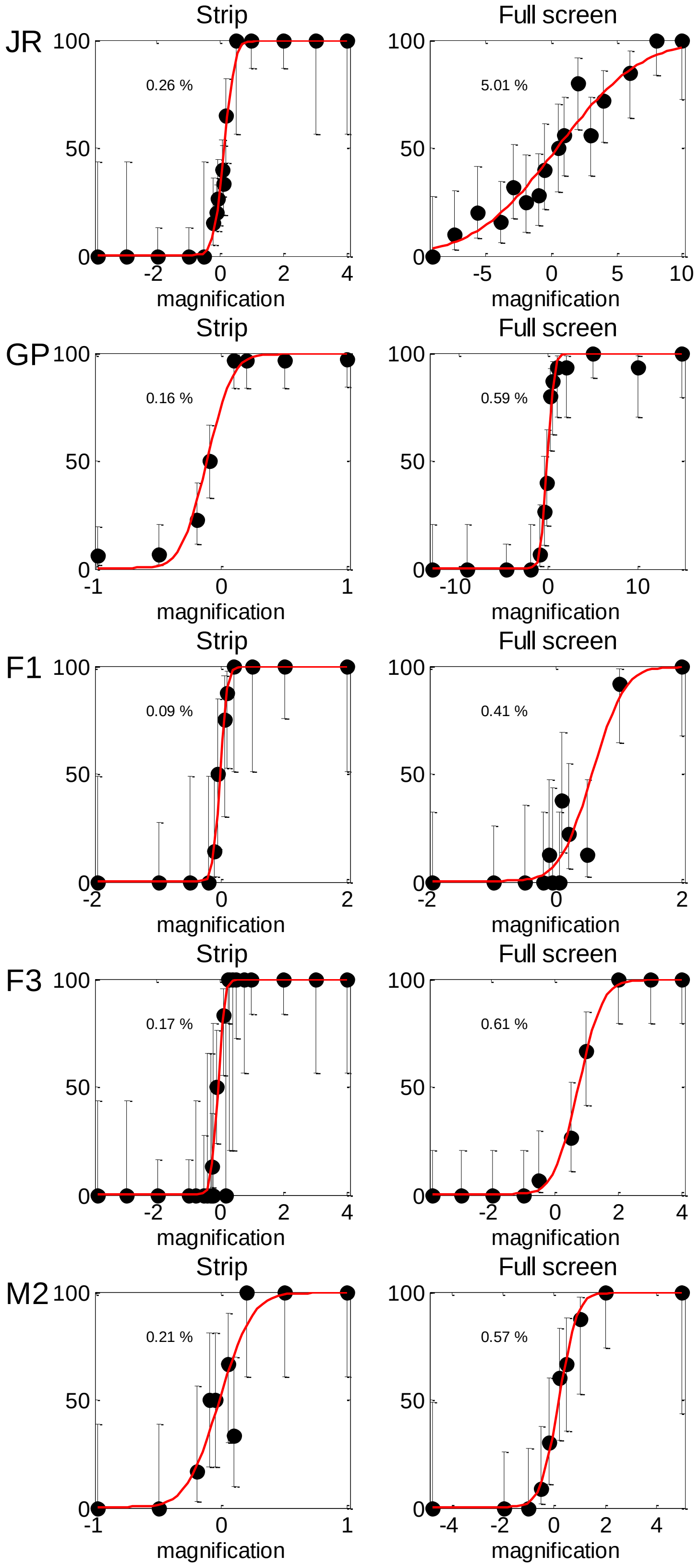

Figure S6. All data collected from control subjects in the disparity-defined slant discrimination task, short durations.

Experiment 3.1: Transparent rotating cylinder

The angular frequency of the cylinder was F=0.2 rotations/second. A dot which started from the edge of the cylinder at time t=0, subsequently moved with horizontal position $X\cos(2\pi Ft)$ and disparity $D\sin(2\pi Ft)$. The total width of the cylinder was $2X$=320 pixels=6°. The maximum disparity D was varied from trial to trial. The horizontal axes in **Error! Reference source not found.** show $2D$. This is the relative disparity between front and back surfaces, or equivalently the absolute disparity of one surface when the subject is fixating on the other.

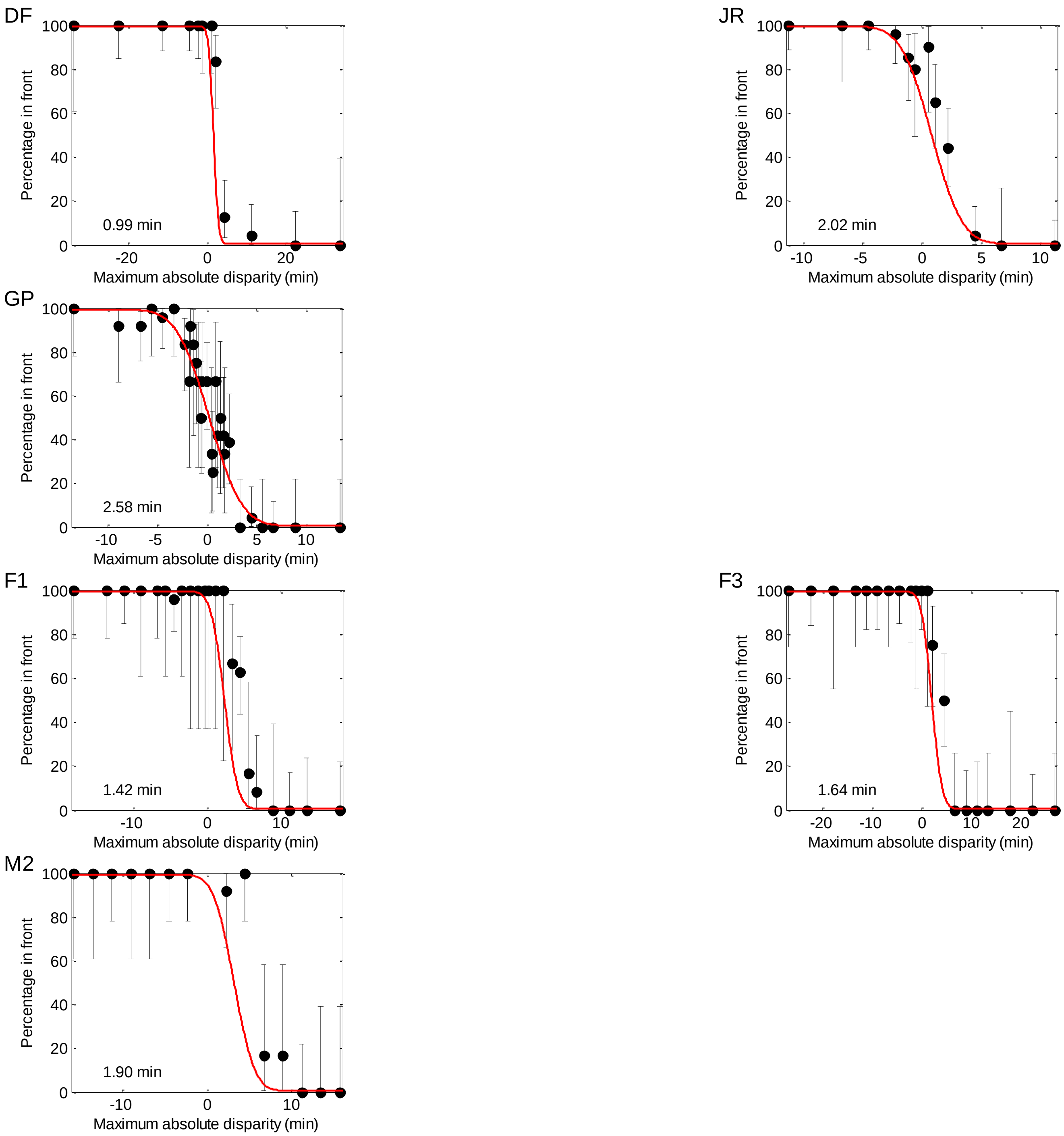

Figure S7. Individual psychometric functions for the rotating cylinder experiment. The horizontal axis shows the maximum relative disparity between the front and back surfaces of the cylinder. The numbers at the bottom left of each panel are the fitted thresholds.
